# Comprehensive analyses of telomerase component DKC1 and its association with clinical, molecular and immune landscapes in uterine corpus endometrial carcinoma

**DOI:** 10.3389/fcell.2025.1592135

**Published:** 2025-05-19

**Authors:** Chenxi Sun, Xu Liu, Tiantian Liu, Chenliu Fan, Yang Jiang, Binggen Li, Huiyang Yuan, Chengyun Zheng, Dawei Xu

**Affiliations:** ^1^ Department of Hematology, The Second Hospital of Shandong University, Jinan, China; ^2^ Department of Pathology, Qilu Hospital of Shandong University, Jinan, China; ^3^ R&D Department, Weihai Zhengsheng Biotechnology Co., Ltd., Weihai, China; ^4^ Department of Urology, Qilu Hospital of Shandong University (Qingdao), Qingdao, China; ^5^ Department of Medicine, Bioclinicum, Karolinska Institutet and Karolinska University Hospital Solna, Stockholm, Sweden

**Keywords:** DKC1, endometrial carcinoma, prognostic factor, tumor microenvironment, T Cell exclusion, telomerase

## Abstract

**Background:**

Telomerase activation is essential to malignant transformation and progression including uterine corpus endometrial carcinoma (UCEC), while telomerase co-factor DKC1-mediated RNA pseudouridylation is required for functional telomerase by stabilizing telomerase RNA component (TERC) and its upregulation occurs in many cancers. Surprisingly, there is only one publication studying DKC1 in UCEC, which shows its significant downregulation.

**Objective:**

DKC1 expression, its role in the UCEC molecular pathogenesis and clinical implications were comprehensively investigated.

**Methods:**

Thirty UCEC patients were recruited to determine DKC1 expression in both tumors and non-tumorous endometrial tissues (NT) using immunohistochemistry. Four UCEC cohorts from TCGA and GSE datasets were analyzed for DKC1 expression and its impacts on clinic-pathological, molecular, genomic and immune landscapes.

**Results:**

Immunohistochemistry analyses showed significantly increased DKC1 expression in UCEC tumors than in NTs and its highest level was observed in high-grade tumors. For the TCGA cohort, DKC1 mRNA and protein levels increased significantly in tumors compared with that in NTs. DKC1 mRNA levels positively correlated with TERC and telomerase activity. Higher DKC1 expression predicted shorter patient overall and progression-free survival. DKC1 copy number alterations were frequent in UCEC tumors. Estrogen treatment of UCEC cells upregulated DKC1 expression while medroxyprogesterone inhibited its expression. DKC1-high UCEC tumors exhibited hyperproliferation, increased stemness and epithelial-mesenchymal transition, accompanied by significantly higher aneuploid, homologous recombination deficiency and micro-satellite instable scores, and higher frequencies of cancer driver aberrations. Lower immune scores were observed in DKC1-high tumors as assessed by ESTIMATE algorithm. Tumor Immune Dysfunction and Exclusion (TIDE) analyses revealed robustly higher TIDE scores featured with T Cell exclusion in DKC1-high tumors, and consistently, the diminished trafficking of immune cells into tumor tissues and substantial declines in immune cell infiltration were shown in these tumors. Moreover, DKC1-high tumors exhibited poor response to immune checkpoint inhibitor (ICI)-based immunotherapy. These observations were validated by the findings obtained from other datasets.

**Conclusion:**

The present findings unravel genomic alteration- and sex hormone-mediated dysregulation of the telomerase cofactor DKC1 in UCEC tumors, and its upregulation participates actively in the UCEC pathogenesis through tumor-intrinsic and extrinsic mechanisms. DKC1 assessment is useful for patient prognostication and personalized interventions.

## 1 Introduction

Uterine corpus endometrial carcinoma (UCEC) or endometrial carcinoma (EC), derived from the uterus endometrial columnar epithelium, is the commonest malignancy in the female reproductive tract (Gu et al., 2021; Sung et al., 2021; Crosbie et al., 2022). During the past years, the incidence of cervical cancer has significantly dropped, however, the UCEC diagnosis has doubled, and there were more than 400,000 new cases worldwide in 2020 (Gu et al., 2021; Sung et al., 2021; Crosbie et al., 2022). From the pathogenesis point of view, UCECs are roughly classified into the following two types: Type I UCEC is predominant (80% or more) and largely caused by the hyper-activity of an estrogen signaling and characterized by endometrioid histology (Rodriguez et al., 2019; Gu et al., 2021; Crosbie et al., 2022), whereas type II (accounting for approximately 20% of all UCECs) are usually high-grade, estrogen-unrelated and featured with serous histology (Gu et al., 2021; Crosbie et al., 2022). Outcomes are much better for patients with type I than those with type II UCEC, but a small fraction of type I patients will develop aggressive diseases (Crosbie et al., 2022). Thus, it is unmet demand to search for reliable predictors for patient categorization, especially for type I UCECs with progression potential, thereby identifying high-risk patients for active surveillance and achieving precision medicine for outcome improvement. To this end, a panel of clinical and pathological variables have long been developed to stratify progression risk and outcomes, however, there exist certain limitations (Crosbie et al., 2022). Recent advances and application in next-generation sequencing and other omics technologies have led to profound insights into UCEC pathogenesis and provided the basis for molecular classifications of UCECs (Cancer Genome Atlas Research et al., 2013; Abdulfatah et al., 2019). Such molecular subtyping combined with clinical phenotypes have significantly improved the robustness of UCEC prognostication. Despite so, because of the heterogenous property of UCECs, identifying more reliable biomarkers is required to stratify patient risk for even better personalized interventions.

UCEC tumors or cells, like all other malignancies, undergo infinite proliferation, which is known to be attributed to telomerase activation (Hapangama et al., 2017; Alnafakh et al., 2019; Yuan et al., 2019; Perez-Lopez et al., 2023). Mechanistically, telomerase adds telomeric DNA sequences to chromosome ends and prevents telomere shortening, thereby overcoming the senescence barrier mediated by critically shortened or dysfunctional telomeres (Hapangama et al., 2017; Alnafakh et al., 2019; Hao et al., 2023; Perez-Lopez et al., 2023). Telomerase is a multi-unit complex, and although its core enzyme is composed of a catalytic component telomerase reverse transcriptase (TERT) and internal telomerase RNA template (TERC) (Roake and Artandi, 2020), other accessory or co-factors in the complex are required for fully functional telomerase, too (Venteicher et al., 2008; Roake and Artandi, 2020; Wang et al., 2023). Indeed, the aberrant expression and/or function of telomerase co-factors are widespread in many kinds of human cancer and significantly promote telomerase activation (Wang et al., 2023). For example, DKC1, in a pseudouridylation enzyme complex associated with TERT and TERC, is required for *in vivo* telomerase function, and its mutations or absence leads to diminished telomerase activity, accelerated telomere erosion and onset of telomere pathology (Vulliamy et al., 2001). Therefore, to fully understand telomerase biology, telomere maintenance and their clinical implications in UCEC, we need to study not only telomerase core components (TERT and TERC), but also telomerase co-factors. However, the majority of telomerase analysis in UCECs have been focused on TERT and TERC (Boggess et al., 2006; Zhou et al., 2013; Hapangama et al., 2017; Alnafakh et al., 2019; Perez-Lopez et al., 2023; Praiss et al., 2023), and there exist only one report about DKC1 in UCEC, and unexpectedly, DKC1 expression was observed to be downregulated in UCEC tumors, especially in aggressive ones (Alnafakh et al., 2021), which are in sharp contrast to the results obtained from other cancer types (Liu et al., 2012; Guerrieri et al., 2020; Hou et al., 2020; Richards et al., 2020; Kan et al., 2021; Mourksi et al., 2023; Wang et al., 2023; Yuan et al., 2023). In the present study, we sought to comprehensively investigate the role of dysregulated DKC1 in UCEC pathogenesis by addressing their association with telomere maintenance, genomic landscape, aberrant molecular signaling pathways, immune microenvironment and clinical significance.

## 2 Materials and methods

### 2.1 UCEC tumors, patients and immunohistochemistry (IHC)

Thirty UCEC patients who underwent surgical operation at Qilu Hospital of Shandong University were included (Qilu cohort), and tumor tissues were paraffin embedded. For IHC staining, tissues on slides were deparaffinized and rehydrated followed by antigen retrieval. Endogenous peroxidase was deactivated by hydrogen peroxide. Slides were blocked using 10% goat serum and incubated with a DKC1 polyclonal antibody (Cat 25420-1-AP, Proteintech, Rosemont, IL) for 2 h at room temperature. After incubation with the secondary antibody (ZSGB Biotechnology, Beijing, China) for 30 min at room temperature, DAB staining (Thermo Fisher Scientific) was applied to visualize the antigen–antibody binding. For each slide, a total of 200 cells were counted, and the scores (0, I, II and III) were calculated based on DKC1 positive cells and staining intensity. Patient clinical data were listed in Supplementary Table S1. The study was approved by the Ethics Committee of Shandong University Second Hospital (#KYLL2024738).

### 2.2 TCGA and other dataset-derived UCEC cohorts, clinic-pathological, and sequencing data processing

**FIGURE 1:**
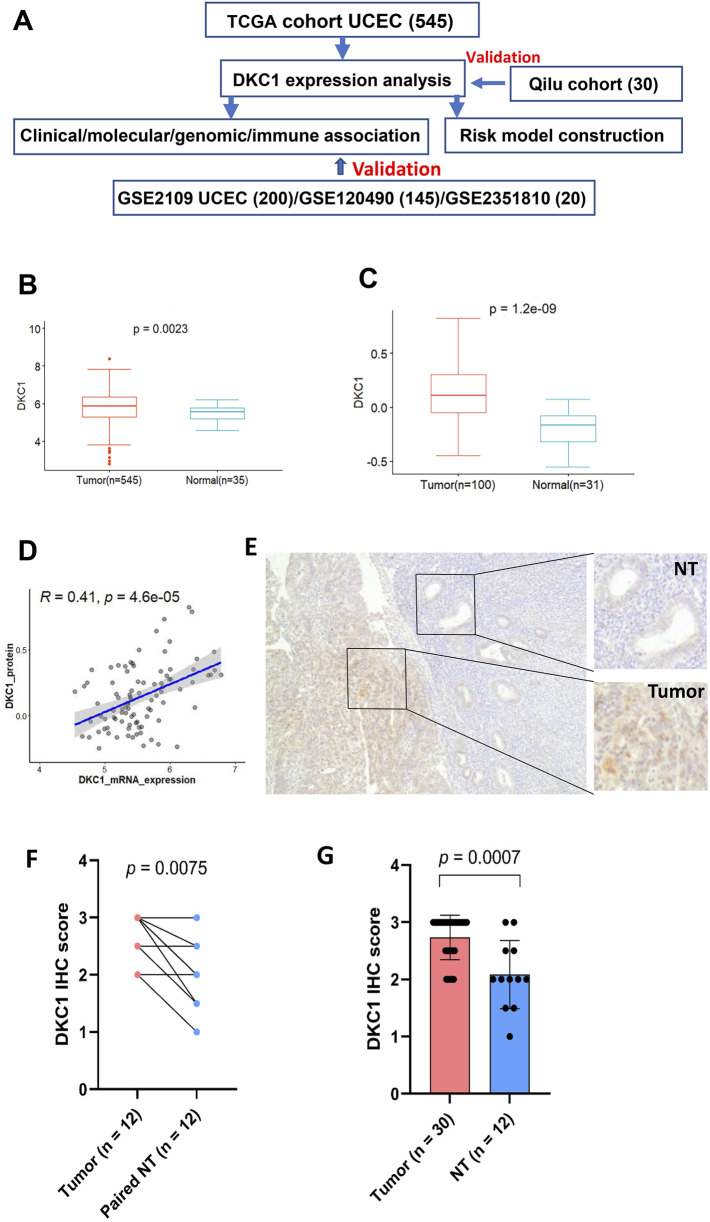
DKC1 expression is upregulated in UCEC tumors **(A)** The flow chart of the study **(B)** The levels of DKC1 mRNA [log2 (TPM+1)] were evaluated and compared between 545 tumors and 35 non-tumorous endometrial tissues in the TCGA UCEC cohort **(C)** The DKC1 protein levels (Z-value) were evaluated and compared between 100 tumors and 31 non-tumorous endometrial tissues in the CPTCA UCEC cohort **(D)** The significantly positive correlation between mRNA and protein levels of DKC1 (Z-value) based on **(B)** and **(C)** results **(E–G)** The upregulation of DKC1 expression in UCEC tumors from the Qilu cohort, as determined using immunohistochemistry (IHC). The representative IHC images in **(E)** showed stronger DKC1 staining in tumors than in adjacent normal glands. Magnifications: ×40 **(F)** Comparison of IHC scores between tumors and adjacent normal glands in 12 paired samples **(G)** Comparison of IHC scores in all 30 tumors with 12 normal gland-containing samples.

We analyzed 4 UCEC cohorts from the Cancer Genome Atlas (TCGA), GSE2109, GSE120490 and GSE2351810 (Figure 1A). The TCGA cohort of UCEC contains 545 patients with 545 tumors and 35 non-tumorous (NT) endometrial specimens (Cancer Genome Atlas Research et al., 2013; Kandoth et al., 2013). Clinical and pathological information data was downloaded from https://gdc.cancer.gov/ (while mutation and copy number were downloaded from https://www.cbioportal.org/) in June 2023. RNA sequencing results of those tumors were downloaded simultaneously, and mRNA abundances were expressed as Transcripts Per Million (TPM) or log2 (TPM+1). DKC1 protein expression data were obtained from Clinical Proteomic Tumor Analysis Consortium (CPTAC) (http://ualcan.path.uab.edu/index.html) (Dou et al., 2020). GSE2109 cohort, derived from the expO dataset, included 200 tumors from UCEC patients and standardized microarray data were downloaded at http://www.ncbi.nlm.nih.gov/geo/in Oct. 2023. Microarray data in GSE120490 (Casablanca et al., 2022) and GSE2351810 (Mhawech-Fauceglia et al., 2011) were downloaded from the above site in Aug. 2024.

Microarray data of estradiol- or Medroxyprogesterone acetate (MPA)-treated Ishikawa cells (UCEC-derived) were downloaded from http://www.ncbi.nlm.nih.gov/geo/with accession numbers GSE11869 (Naciff et al., 2009) and GSE29435 (van der Horst et al., 2012), respectively, in Oct. 2023.

### 2.3 Development of a predictive nomogram for progression-free survival (PFS)

Cox regression analysis was performed to determine the impact of DKC1 expression and clinical variables on PFS, and we then constructed a predictive nomogram that included DKC1, stage and age to predict PFS. Predicted PFS of the nomogram against observed ones was plotted using the calibration curve. All nomograms and assessments of their predicative powers were made using R package “regplot” (rms). In addition, time-dependent ROC curves and area under curves (AUCs) were used to estimate the accuracy of identified PFS predictors in UCEC patients.

### 2.4 Kyoto encyclopedia of genes and genomes enrichment analyses (KEGG) and gene set enrichment analysis (GSEA)

Reference gene signatures for KEGG analysis were downloaded from https://www.gsea-msigdb.org/gsea/index.jsp (h.all.v2023.2). “Hs.symbols.gmt’ and ‘c2. cp.kegg_legacy.v2023.2. Hs.symbols.gmt’). Differences in KEGG pathways between DKC1-high and low expression groups were determined using GSEA (version 4.3.2). Adjusted *P* value < 0.05 and *FDR* < 0.05 were regarded as significantly different pathways. Heatmap was made using R package “Complex”.

### 2.5 Copy number alteration (CNA), aneuploidy score, homologous recombination deficiency (HRD), tumor mutation burden (TMB) and mitochondrial DNA copy number analysis

Somatic CNAs were downloaded from https://www.cbioportal.org/. CNA plots were made using R package ‘ComplexHeatmap’. Aneuploidy scores were the sum total of altered (amplified or deleted) chromosome arms (AM Taylor et al., 2018). HRD scores were from Knijnenburg et al. (Knijnenburg et al., 2018). TMB is defined as the number of non-silent mutations per million bases and calculated using r package. Mitochondrial DNA copy number of UCEC was obtained from Reznik E et al. (Reznik et al., 2016).

### 2.6 Analyses of immune environments in UCEC tumors

ESTIMATE algorithm, Tumor Immune Dysfunction and Exclusion (TIDE), Cancer immune cycle or tracking immune phenotypes (TIP) and Cancer Immune Atlas (TCIA) were used to characterize immune environment landscape and sensitivity to immune checkpoint inhibitors (ICIs) in UCEC tumors. TIDE score is calculated based on myeloid-derived suppressor cell (MDSC), macrophage M2, T Cell Dysfunction and Exclusion (Jiang et al., 2018). The TIDE score in TCGA UCEC cohort was directly downloaded from http://tide.dfci.harvard.edu/. TIP analysis was performed based on Xu et al. at https://github.com/dengchunyu/TIP (Xu et al., 2018). TCIA was carried out to estimate potential response to ICIs and the immunophenoscore (IPS), a quantitative index to evaluate the cancer-immunity cycle (CIC) efficacy, in each TCGA UCEC tumor was downloaded from TCIA website (https://tcia.at/home). In addition, differences in expression of MHC gene sets (CANX, CALR, PDIA3, ERAP2, B2M, HLA-A, ERAP1, TAPBP, PSMB8, PSMB9, TAP1, NLARC5, TAP2, HLA-C, HLA-B) (Lauss et al., 2017) were compared.

### 2.7 Analyses for proliferation, cell cycle score, stemness, EMT, telomerase or EXTEND scores and telomere length

UCEC tumor proliferation was evaluated using expression levels of Ki-67 and cell cycle scores, respectively. Cell cycle score was calculated based on single sample GSEA (ssGSEA) using the following gene panel: CDK2, CDK4, CDK6, BUB1B, CCNE1, POLQ, AURKA, MKI67 and CCNB2 (Hao et al., 2023). Stemness score was calculated based on ssGSEA of 109 gene signatures as described (Miranda et al., 2019). EMT scores were calculated based on the following gene expression: VIM, CDH2, FOXC2, SNAI1, SNAI2, TWIST1, FN1, ITGB6, MMP2, MMP3, MMP9, SOX10, GCS, CDH1, DSP and OCLN (Gibbons and Creighton, 2018). Telomerase score was calculated according to expression levels of 10 telomerase components (TERT, TERC, DKC1, TCAB1, NHP2, GAR1, NOP10, RUVBL1 and 2, and NVL) as described (Wang et al., 2023). EXTEND algorithm was used to estimate telomerase activity using a 13 gene signature, according to Noureen et al. (Noureen et al., 2021). Telomere length data in UCEC tumors and blood cells were obtained from (Barthel et al., 2017).

### 2.8 Statistical analysis

Statistical analyses were performed using R package version 4.3.0. According to data distributions, Student’s t-test, Wilcox and K-W sum tests, and Chi^2^-or Fish exact tests were used for analysis. Correlation between gene expression levels was evaluated by Pearson coefficient correlation (Spearman’s Rank-Order Correlation coefficient). Kaplan-Meier analysis with log-rank test was carried out to evaluate overall survival (OS) and PFS among groups. The effect of various quantitative variables on OS and PFS was measured by univariate and multivariate Cox regression analyses. Multivariate Analysis of Variance (MANOVA) was used to assess whether DKC1-related molecular/genomic features were dependent on stages and grades. *P* < 0.05 were considered as statistically significant.

## 3 Results

### 3.1 DKC1 expression in UCEC tumors and matched non-tumorous specimens

The flow chart of the present study was shown in Figure 1A. We first analyzed 545 tumors and 35 matched non-tumorous specimens (NTs) in the TCGA UCEC cohort for their DKC1 expression. Compared to NTs, DKC1 mRNA levels increased significantly in UCEC tumors (NT vs. UCEC, *P* = 0.002) (Figure 1B). Among those specimens, 31 NTs and 100 tumors were also examined for DKC1 protein expression in the CPTAC project (Dou et al., 2020), and their protein levels were significantly higher in UCEC tumors (NT vs. UCEC, *P* = 1.20E-10^9^) (Figure 1C). DKC1 mRNA and protein levels were positively correlated with each other (Figure 1D). Thus, DKC1 expression is aberrantly upregulated at both mRNA and protein levels in UCEC tumors.

### 3.2 Upregulation of DKC1 expression in qilu cohort of UCEC tumors assessed by IHC

The TCGA UCEC cohort analyses above show the significant upregulation of DKC1 expression at both mRNA and protein levels in tumors. However, a recent study reported reduced DKC1 expression compared to their NT counterparts, as determined using IHC, which was opposite to the TCGA result. To address these differences, we also employed IHC to examine 30 primary UCEC samples for their DKC1 levels. Among 30 specimens, 12 of them contained normal glands sufficient for score evaluation (Additional Supplementary Table S1). As shown in Figures 1E–G, both tumors and normal glands expressed DKC1, but significantly stronger staining and higher scores were observed in tumors (compared between either paired samples or total ones). Moreover, more abundant DKC1 was observed in tumors with higher grade (Additional Supplementary Table S1).

### 3.3 Association of DKC1 expression with TERC, telomerase activity, clinic-pathological characteristics of UCEC patients

**FIGURE 2:**
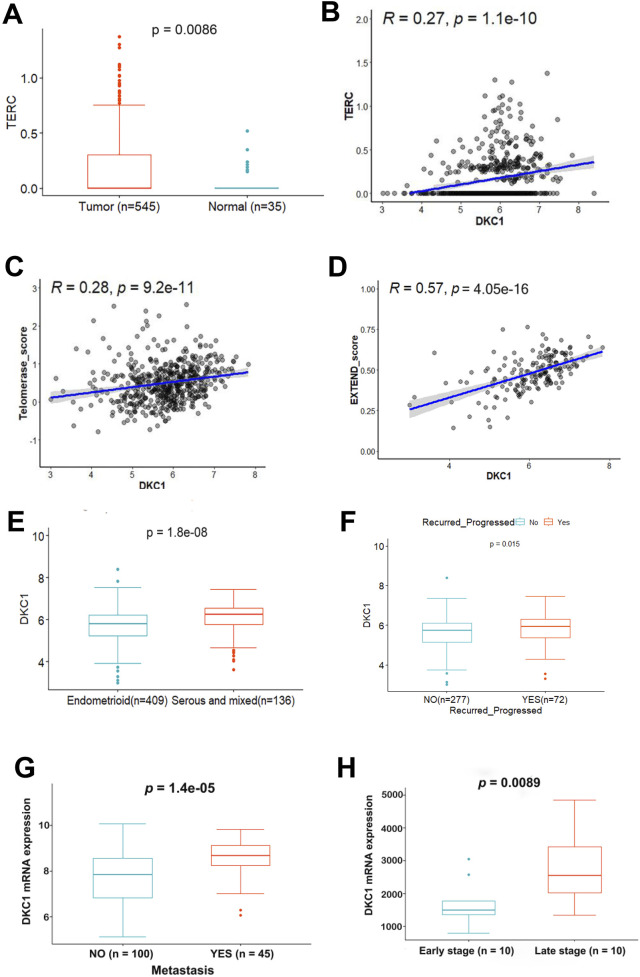
The positive correlation between higher DKC1 expression and TERC, telomerase activity and aggressive UCEC tumors **(A–F)** RNA levels were assessed using log2 (TPM+1). The TCGA cohort of UCEC was analyzed **(A)** Upregulation of TERC expression in UCEC tumors **(B)** The positive correlation between DKC1 and TERC expression **(C, D)** The positive correlation between DKC1 and telomerase activity. Telomerase activity levels were calculated using the telomerase score (Ref. 14) and EXTEND (Ref. 41) algorithms, respectively **(E)** Significantly higher DKC1 expression in serous and mixed types of UCECs **(F)** The association between higher DKC1 expression and higher risk of recurrence **(G)** The association between higher DKC1 expression and higher frequency of metastasis. The GSE120490 UCEC cohort with 145 UCEC patients (100 without and 45 with metastasis) were analyzed **(H)** Significantly higher DKC1 expression (microarray data) in late-stage UCEC tumors from the GSE23518 cohort (with 10 early and 10 late-stage UCECs).

Because DKC1-mediated RNA pseudouridylation is required to stabilize TERC RNA (Angrisani et al., 2014; Yuan et al., 2023), we next compared TERC expression between NTs and UCEC tumors from the TCGA cohort. As expected, TERC expression increased significantly in UCEC tumors (NT vs. UCEC, *P* = 0.0086) (Figure 2A). Moreover, TERC RNA levels were positively correlated with DKC1 expression (Figure 2B). Given the enhanced DKC1 and TERC expression observed in UCEC tumors, while enzymatic activity as a fundamental metric of telomerase, we further determined whether telomerase enzymatic activity was upregulated in such settings. Telomerase activity was assessed using both telomerase score (Wang et al., 2023) and EXTEND algorithms (Noureen et al., 2021). Telomerase score (Figure 2C) correlated with DKC1 levels based on the analysis of 545 tumors. There were 172 tumors with EXTEND score available (Noureen et al., 2021), and the EXTEND score was similarly correlated with the DKC1 expression, which was much stronger than telomerase score (Figure 2D).

We then determined whether there was an association between DKC1 expression and clinic-pathological variables in UCEC tumors. Of note, serous/mixed UCEC tumors exhibited robustly higher levels of DKC1 (Figure 2E) (Table 1). Significantly upregulated DKC1 expression was also observed in advanced stages and grades of tumors (Table 1). Intriguingly, patient BMI was inversely associated with DKC1 (Table 1). Age, diabetes, and hypertension had no impact on DKC1 expression (Table 1). Moreover, in the UCEC cohort studied by Kanton et al. (Kandoth et al., 2013), 72 of 349 patients underwent disease recurrence/progression and their tumors expressed significantly higher levels of DKC1 (Figure 2F).

**TABLE 1:** Clinico-pathological characteristics and association with DKC1 expression in UCEC (TCGA).

Variable	Informative number	DKC1 (mean ± sd)	*P* Value
Age (year) <60 ≥60	179 363	60.295 ± 29.7768 65.7679 ± 36.4737	0.160
Prior tamoxifen administered usage never used Used	355 8	62.4027 ± 30.9375 106.4802 ± 101.7077	0.25
Histology Endometrioid Serous and mixed	409 136	59.9754 ± 34.037 76.0705 ± 32.869	0.001
Stage I + II III + IV	392 153	62.1289 ± 35.1453 68.7645 ± 32.1549	0.008
Grade G1+G2 G3	220 325	50.9843 ± 27.7597 72.7968 ± 35.7367	< 0.001
Diabetes Yes No	113 304	62.6515 ± 36.9142 63.0583 ± 32.3645	0.811
Hypertension Yes No	268 181	61.1491 ± 30.3019 66.1632 ± 38.006	0.266
BMI <25 25-30 >30	95 114 305	75.1001 ± 42.7156 59.4794 ± 27.8472 61.6595 ± 33.3701	0.003

Abbreviations: BMI, Body mass index.

To validate the findings obtained from the TCGA UCEC cohort, we further analyzed three UCEC cohorts from the GSE2109, GSE120490 and GSE2351810. There were two hundred UCEC tumors in the GSE2109 cohort (Additional Supplementary Table S2), and transcriptomic profiling was assessed using microarray. Higher-grade tumors expressed significantly higher levels of DKC1. Increased DKC1 expression was observed in high tumor stages (T3/T4). DKC1 expression was upregulated significantly in serous/mixed tumors. The GSE120490 cohort included 145 UCEC patients among which 100 had no metastasis while 45 underwent metastasis, and significantly higher DKC1 mRNA expression was observed in those tumors with metastasis (*P* = 1.4E-05) (Figure 2G). For the GSE2351810 cohort with 20 UCEC patients, 10 tumors from the late-stage patients expressed significantly higher levels of DKC1 mRNA than did those from early-stage patients (*P* = 0.009) (Figure 2H). Taken together, these results are largely consistent with TCGA data analyses.

### 3.4 Higher DKC1 expression as a prognostic factor for UCEC patient survival

**FIGURE 3:**
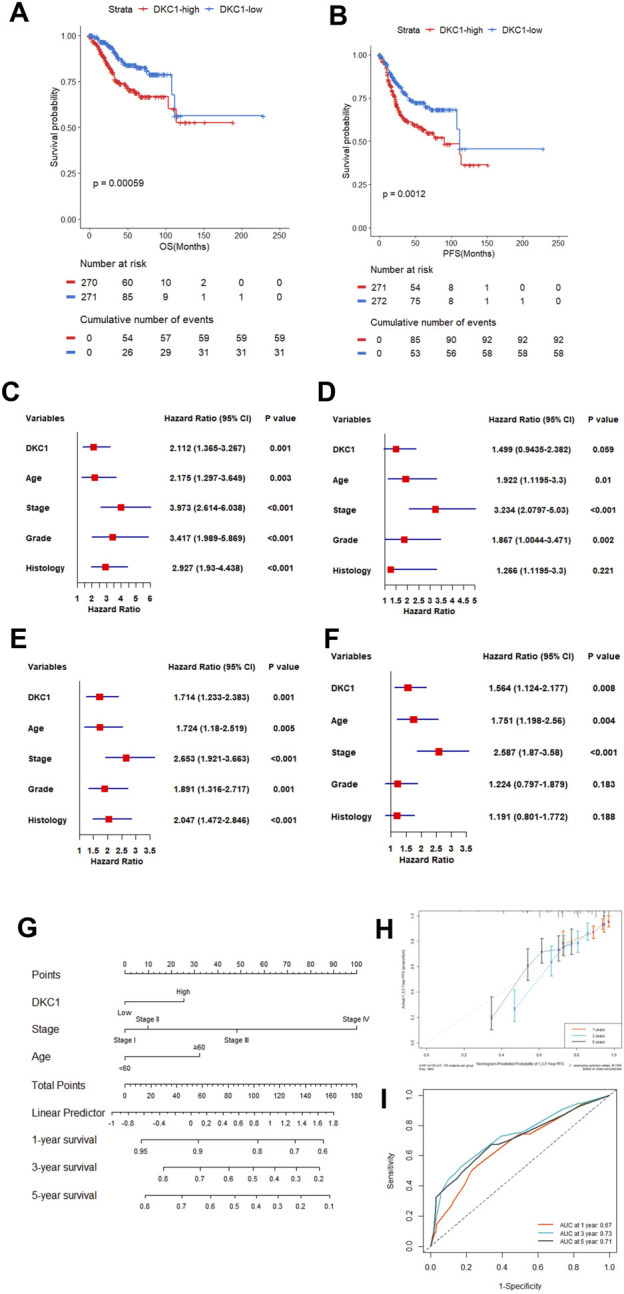
Higher DKC1 expression predicts UCEC patient survival independently. Patients in the TCGA UCEC cohort were categorized into low and high groups based DKC1 mRNA levels in their tumors (median value as the cutoff) **(A, B)** Association between DKC1 expression and overall and progression-free survival (OS and PFS) **(C, D)** Univariate and multivariate COX regression analyses of DKC1 effect on patient OS **(C)** Univariate and **(D)** Multivariate **(E, F)** Univariate and multivariate COX regression analyses of DKC1 effect on patient PFS **(E)** Univariate and **(F)** Multivariate **(G–I)** Nomogram for prediction of UCEC PFS. A total of 349 patients were analyzed by including DKC1 (high vs. low), stage (I/II vs. III/IV) and age (<60 vs. ≥60) **(H)** The accuracy of the nomogram to predict PFS (Prediction curve vs. observed scenario) **(I)** The ROC prediction of PFS. ROC showed AUC values 0.67, 0.73 and 0.71 at 1, 3 and 5 years PFS, respectively. RNA levels were calculated using log2 (TPM+1).

We then sought to determine the impact of DKC1 on OS and PFS in UCEC patients. For this purpose, we categorized the TCGA UCEC patients into high- and low-groups based on their DKC1 levels using median values as cutoffs, and Kaplan-Meier survival analyses were performed. As shown in Figures 3A, B, patients in the DKC1 high-group had significantly shorter OS and PFS than those in the low-group.

Univariate and multivariate COX regression analyses were then performed to determine whether DKC1 served as independent prognostic factors. The univariate COX analyses revealed that higher DKC1 expression, age ≥60, advanced stages (III and IV), higher grades (III and IV) and non-endometrial histology were all significantly associated with shorter OS (Figure 3C), whereas P value for DKC1 was at the borderline (0.059) and histology was no longer significant when the multivariate analyses were carried out (Figure 3D). For PFS, higher DKC1 expression led to significantly shorter survival as unraveled by both univariate and multivariate analyses (Figures 3E,F). Thus, DKC1 is an independent prognostic factor in UCEC.

Based on the multivariate COX analyses, we combined higher DKC1 with patient age (≥60), and advanced stages (III and IV) to establish a nomogram to predict PFS (Figure 3G), which was largely consistent with the observed scenario (Figure 3H). ROC showed AUC values 0.67, 0.73 and 0.71 at 1, 3 and 5 years PFS, respectively (Figure 3I).

### 3.5 Association of DKC1 expression with genomic alterations, sex hormones and telomere length in UCECs

**FIGURE 4:**
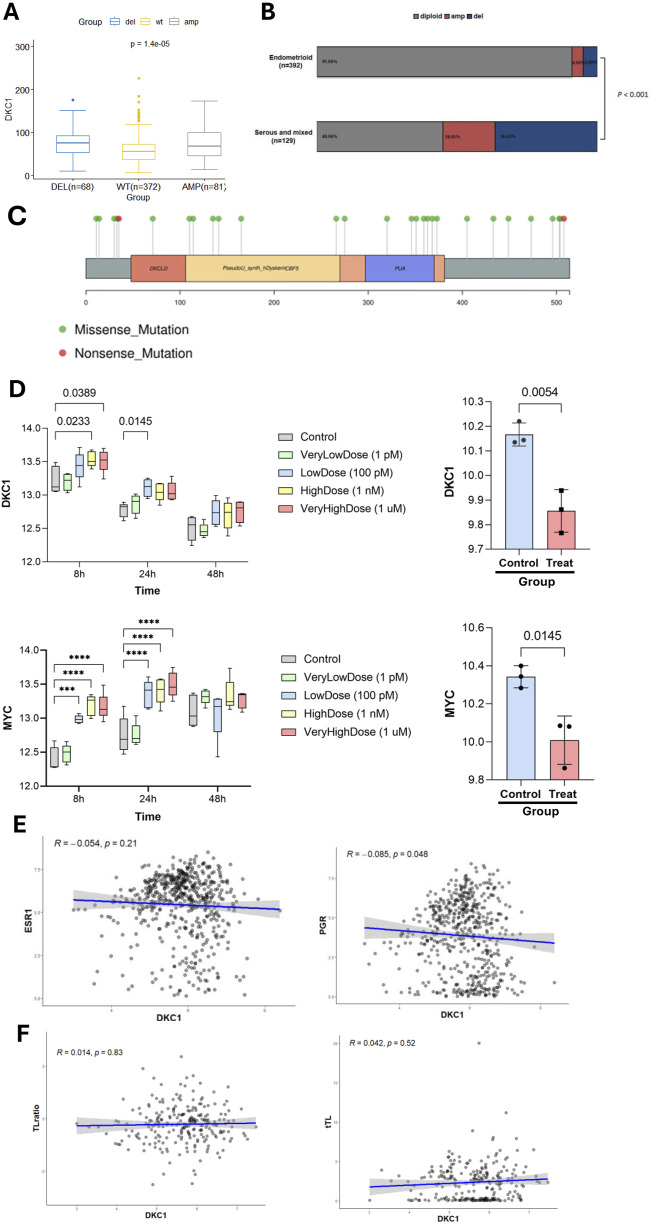
DKC1 expression is regulated by genomic alterations and female sex hormones but not by telomere length in UCEC tumors and cells. The TCGA cohort of UCEC tumors and UCEC-derived cells were analyzed **(A)** Differences in DKC1 expression in UCEC tumors carrying different copy numbers **(B)** Differences in DKC1 copy numbers between endometrial and serous/mixed types of UCEC tumors **(C)** The mutational landscape of the *DKC1* gene in UCEC tumors **(D)** Up- and downregulation of DKC1 (Top panel) and MYC (Bottom panel) mRNA expression in UCEC-derived Ishikawa cells treated by 17 β-estradiol (left) and 1 nM MPA (right), respectively. *** and ****: P < 0.001 and 0.0001, respectively. Three independent experiments were performed **(E)** Correlation between DKC1 and estrogen receptor 1 (ESR1) (left) or PGR (right) expression **(F)** No correlation between DKC1 expression and the ratios of telomere length of UCEC tumors and corresponding patient blood cells. Telomere length of UCEC tumors and corresponding patient blood cells were obtained from reference Barthel FP, et al.

We then sought to probe potential mechanisms underlying dysregulation of DKC1 in UCECs. DKC1 copy number alterations were frequent in UCECs, which included both deletion (68/521, 13%) and gain or amplification (81/521, 16%) (Figure 4A). These alterations were significantly more frequent in serous UCEC tumors (Figure 4B). Intriguingly, tumors with either the gene deletion or gain expressed higher levels of DKC1 mRNA (Figure 4A), suggesting that DKC1 expression was not related to its gene dosages. As germline *DKC1* mutations are known to cause telomere pathology (Vulliamy et al., 2001), we further analyzed their mutations in UCEC tumors. The mutations seemed random, and no mutations took place on known sites that impaired the enzymatic function of DKC1 (Figure 4C).

The analysis of 17 β-estradiol treated UCEC-derived Ishikawa cells showed significant DKC1 upregulation (Figure 4D, top panel left), while MPA inhibited its expression (Figure 4D, top panel right). MYC is an established target gene of estrogen, and its expression was analyzed as a positive control (Figure 4D, Bottom panel). Given the results above, we then analyzed a relationship between their mRNA levels and ESR1 and PGR in UCEC tumors. DKC1 expression was not correlated with ESR1 expression (Figure 4E, left), while with PGR expression inversely (Figure 4E, right).

Because telomere dysfunction is a driving-force for telomerase activation in oncogenesis, we determined whether DKC1 expression was associated with telomere length. As shown in Figure 4F (left panel), DKC1 mRNA levels were not correlated with tumor telomere length. To exclude the age interference, we also calculated ratios of telomere length between patient tumors and normal blood cells, and there was no correlation, either (Figure 4F, right). Similar results were obtained when the correlation between TERT and telomere length was assessed (Supplementary Figure S1).

### 3.6 Identification of molecular features and enriched pathways/hallmarks in DKC1-high UCEC tumors

**FIGURE 5:**
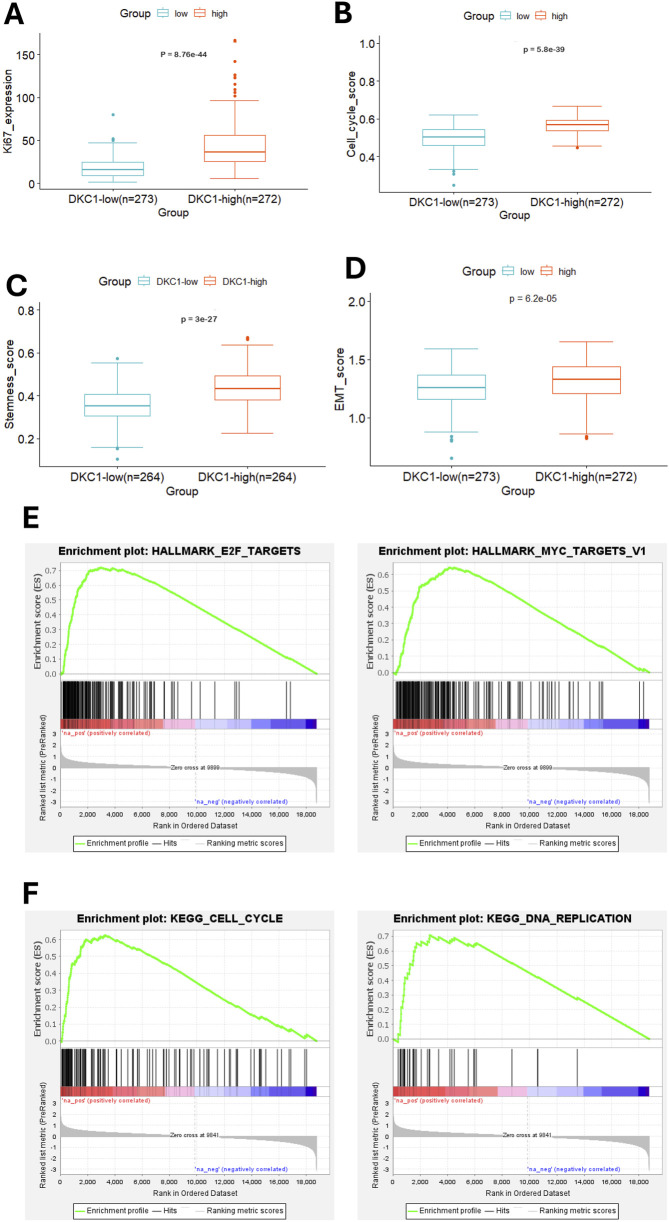
Molecular features and pathway enrichments in DKC1-high UCEC tumors. A total of 545 tumors in the TCGA UCEC cohort were analyzed. Robustly increased Ki67 expression **(A)**, cell cycle score **(B)**, Stemness score **(C)** and EMT score **(D)** in DKC1-high tumors **(E)** The identification of enriched E2F and MYC targets as the hallmarks in DKC1-high tumors by GSEA analysis **(F)** The enriched cell cycle and DNA replication pathways in DKC1-high tumors by KEGG analysis.

To understand mechanisms underlying DKC1-associated poor outcomes, we sought to determine molecular characteristics and pathway enrichments between DKC1-high and low groups. To this end, we first analyzed cell proliferation or cell cycle, stemness and EMT. KI-67 was used as a proliferation marker and its expression was robustly higher in DKC1-high tumors (high vs. low, *P* = 8.76E-10^44^) (Figure 5A). Consistently, DKC1-high tumors exhibited significantly higher cell cycle scores (high vs. low, *P* = 5.80E-10^39^) (Figure 5B). Further comparisons of cancer stemness and EMT scores showed remarkable differences, with enhanced values in DKC1-high tumors (Stemness: high vs. low, *P* = 3.00E-10^27^; EMT: *P* = 6.20E-10^5^) (Figures 5C,D). GSEA analysis for hallmarks showed the following top two enriched pathways for DKC1-high tumors: E2F targets and MYC targets V1 (Figure 5E), whereas the KEGG results revealed Cell cycle and DNA replication as the top ones (Figure 5F).

### 3.7 DKC1 association with UCEC molecular subtypes and genomic alterations

**FIGURE 6:**
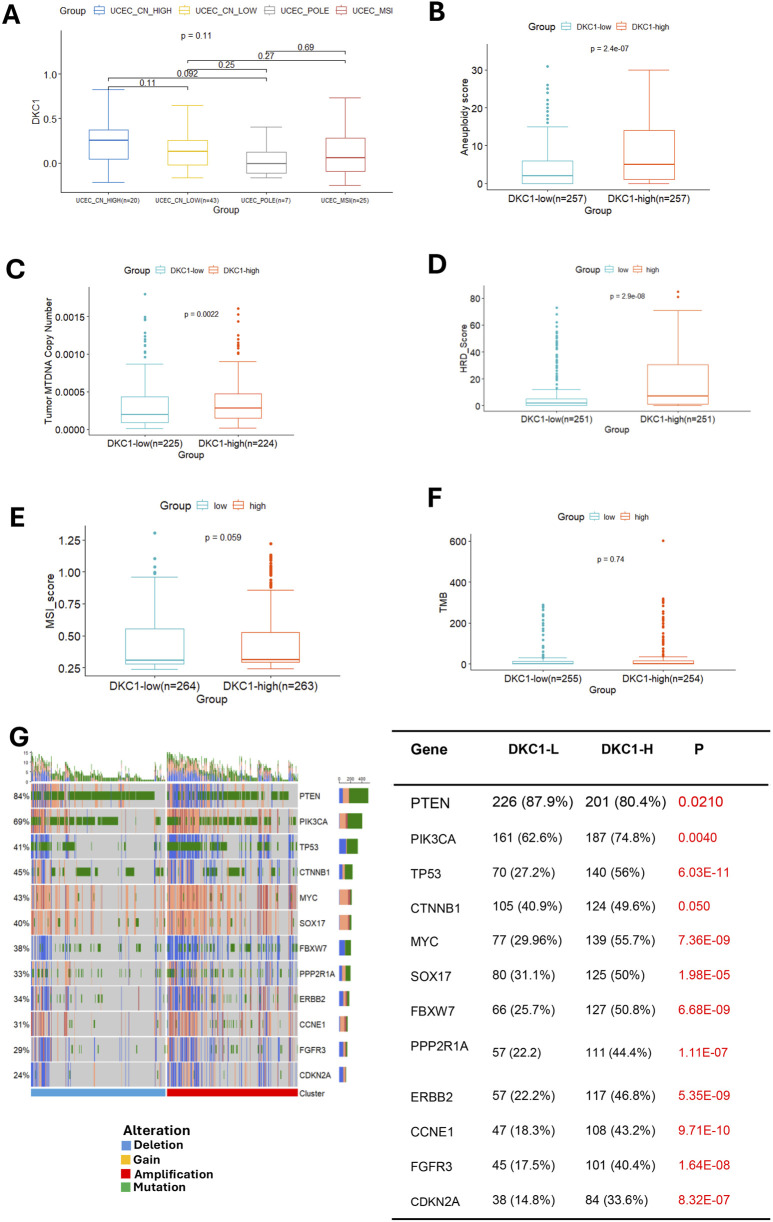
DKC1 expression is associated with UCEC molecular subtypes and genomic aberrations. A total of 545 tumors in the TCGA UCEC cohort were analyzed **(A)** The association between DKC1 mRNA expression and molecular subtypes of UCECs **(B–F)** Comparisons of genomic alterations between DKC1-low and high tumors: Aneuploidy scores **(B)**, mitochondrial DNA (MTDNA) copies **(C)**, HRD **(D)**, MSI **(E)** and TMB **(F) (G)** Different frequencies of genomic alterations in important UCEC driver genes between DKC1-low and high tumors.

UCEC tumors are categorized into the following four molecular subtypes: CN-high, CN-low, microsatellite instability (MSI) (hypermutated) and POLE (ultramutated) (Cancer Genome Atlas Research et al., 2013). We thus assessed the relationship between DKC1 expression and molecular subtypes in ECs. A total of 507 patients with molecular subtype information were available, and DKC1 mRNA expression differed significantly among those four subtypes (Figure 6A). The lowest levels of DKC1 were observed in CN-low tumors and there were significant differences compared to CN-high (*P* = 3.3E-13), POLE (*P* = 6.4E-05) or MSI (*P* = 0.0025). DKC1 expression within CN-high, POLE and MSI subtypes was not different significantly (Figure 6A).

We then compared differences in genomic alterations between DKC1-high and low tumors. At global genomic levels, aneuploid score, mitochondrial DNA copy numbers and HRD scores were significantly higher in DKC1-high tumors (Figures 6B–D), while there were no significant differences in MSI and TMB (Figures 6E,F).

Although there was no difference in TMB at the global level between DKC1-high and low tumors, our further analyses unraveled more frequent alterations of UCEC drivers in DKC1-high tumors (Figure 6G left). These aberrant genes included both oncogenic drivers and tumor suppressors. DKC1-high tumors exhibited higher rates of activating-mutations or copy gains of the oncogenic drivers (PIK3CA, MYC, SOX17, PPP2RA1, ERBB2, CCNE1, FGFR3 and CTNNB1) whereas more frequent inactivating mutations or copy loss of the tumor suppressors (TP53, PTEN, CDKN2A, and FBXW7) (Figure 6G right).

Because DKC1-high tumors were more frequent in advanced stages and grades, we performed the MANOVA test to see whether the DKC1-related molecular/genomic features were independent of stages/grades or interdependent with them. Cell cycle, stemness, EMT, Aneuploidy, and HRD scores, Ki67 expression and tumor MTDNA copies were included. As shown in Supplementary Table S3, cell cycle scores were dependent on all three variables: DKC1, advanced stages and grades, and their interaction as well, whereas HRD scores were significantly dependent on the interaction between advanced stages and grades. All remaining features were stage/grade independent.

### 3.8 Immuno-exclusive microenvironments in DKC1-high UCEC tumors

DKC1 or other telomerase-related gene mutations are known to induce accelerated telomere shortening, thereby leading to genomic instability that consequently increases cancer susceptibility, however, a recent observation shows that compromised immune surveillance rather than genomic instability is attributable to cancer development (Schratz et al., 2023). Therefore, we determined whether DKC1 dysregulation affects UCEC immune landscape.

**FIGURE 7:**
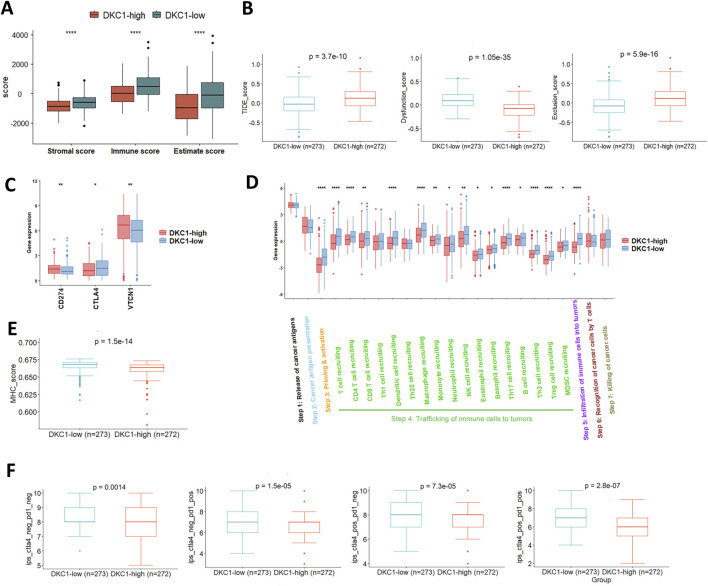
Identification of defective anti-tumor immunity and immunoexclusion microenvironments in DKC1-high tumors. A total of 544 tumors in the TCGA UCEC cohort were analyzed **(A)** Differences in immune, stromal and estimate scores between DKC1-high and low tumors, as determined using ESTIMATE analysis **(B)** TIDE analyses for comparison between DKC1-high and low tumors **(C)** CD274, CTLA4 and VTCN1 expression in DKC1-high and low tumors **(D)** Cancer immune cycle analyses of DKC1-high and low tumors. *, ** and ***: P < 0.05, 0.01 and 0.001, respectively **(E)** Differences in MHC scores between DKC1-high and low tumors **(F)** Prediction of DKC1-high and low tumors to immune checkpoint inhibitor sensitivity. Higher DKC1 expression is associated with lower sensitivity to immune checkpoint inhibitors.

UCEC tumors were scored using ssGSEA to quantify the activity, enrichment level and function of immune cells in each sample, and then categorized based on their DKC1 expression. The ESTIMATE algorithm was used to calculate the stromal, immune and ESTIMATE scores of UCECs. The ESTIMATE, immune and stromal scores in DKC1-high tumors were all significantly lower (Figure 7A). We then performed TIDE analyses in UCECs. As shown in Figure 7B, DKC1-high tumors exhibited significantly higher TIDE scores and more specifically, they were characterized by robustly higher T Cell exclusion scores coupled with lower levels of dysfunction (Figure 7B). To validate the findings above, we further compared VTCN1, PD-L1 and CTLA-4 expression between DKC1-high and low tumors, because immunoexclusion was frequently characterized by upregulated VTCN1 expression and low or unchanged PD-L1 and CTLA-4 levels (Lu et al., 2022). Indeed, significantly higher VTCN1 levels were observed in both DKC1-high tumors (Figure 7C).

The tracking tumor immunophenotype (TIP) was previously developed to analyze a 7-step cancer immune cycle, namely,: release of cancer cell antigens (step 1), cancer antigen presentation (step 2), priming and activation (step 3), trafficking of immune cells to tumors (step 4), infiltration of immune cells into tumors (step 5), recognition of cancer cells by T Cells (step 6), and killing of cancer cells (step7) (Xu et al., 2018). To further evaluate activity of anticancer immunity associated with DKC1 expression in UCECs, we carried out TIP analysis (Xu et al., 2018). The most significant defects in DKC1-high tumors were highly reduced immune cell priming and activation (step 3), and the diminished trafficking of immune cells into tumor tissues (step 4). Of note, the difference in T Cell recruitment was robust at step 4, which consequently led to substantial declines in immune cell infiltration in DKC1-high tumors (step 5) (Figure 7D). These results are highly accordant with the TIDE analysis. To determine whether impaired TIP resulted from MHC defects in DKC1-high tumors, we compared the MHC score between DKC1-high and low tumors, and significantly lower MHC scores were observed in DKC1-high tumors (Figure 7E). Finally, Cancer Immune Atlas (TCIA) analyses were conducted to predict potential response to immune checkpoint inhibitors (ICIs) Based on tumor IPS. DKC1-high tumors exhibited poorer efficacy in anti-PD-L1 or anti-CTLA-4 treatments (Figure 7F).

## 4 Discussion

Human telomeres, composed of TTAGGG repetitive sequences and their associated factors or shelterin proteins, undergo progressive shortening in normal somatic cells with their *in vitro* proliferation or *in vivo* aging; and when telomere length reaches a critical point and becomes dysfunctional, replicative senescence or apoptosis are induced (Yuan et al., 2019). During the UCEC pathogenesis, stabilizing telomere length by telomerase activation is a prerequisite for malignant transformation of endometrial cells (Hapangama et al., 2017; Alnafakh et al., 2019). In the past decades, most studies have been focused on TERT and TERC, two core enzyme components in the telomerase complex (Yuan et al., 2019). To thoroughly understand the role for telomerase and telomere maintenance in UCEC development and progression, we performed comprehensive analyses of two telomerase cofactors DKC1 in UCEC tumors with the following findings: (1) The genomic alterations and dysregulated expression of DKC1 are widespread in UCEC tumors; (2) Higher DKC1 expression at either mRNA or protein level is associated with aggressive UCEC and significantly shorter patient survival; (3) DKC1-high tumors are characterized by frequent UCEC-driver alterations, aggressive phenotypes and impaired anti-cancer immunity.

So far, there has been only one publication investigating DKC1 in UCECs. Hapangama et al. analyzed their UCEC cohort with 109 patients and they observed that higher levels of DKC1 protein expression were associated with better outcomes, as assessed using immunohistochemical staining (Alnafakh et al., 2021). The downregulation of DKC1 expression started in precursor lesions of endometrium and was even more remarkable with the development and progression of UCEC (Alnafakh et al., 2021). These results are contrary to our present analysis. In the TCGA cohort, both mRNA and protein levels of DKC1 were robustly upregulated in UCEC tumors, and their high expression independently predicted shorter patient survival. Our IHC analyses of 30 UCEC-derived tissues further demonstrated significant upregulation of DKC1 in tumors compared to adjacent normal endometrial glands. Three GSE UCEC cohort analyses showed that higher DKC1 expression was observed in aggressive tumors. These findings collectively demonstrate the aberrant upregulation of DKC1 expression in UCEC tumors.

The molecular and pathway analysis further supports the driving-role for DKC1 in UCEC pathogenesis. First, DKC1-high tumors are characterized by hyper-proliferation, and robustly increased stemness and EMT scores. Second, GSEA analysis for KEGG revealed enriched cell cycle and DNA replication pathways in DKC1-high tumors. For hallmark analyses, DKC1-high tumors are overrepresented by E2F and MYC targets. Finally, the genomic alterations that activate oncogenes while inactivate tumor suppressors are much more frequent in DKC1-high tumors, for instance, significantly higher percentages of TP53 inactivation in these tumors. In a mouse UCEC model, defective telomere maintenance is required for TP53 inactivation-mediated disease progression (Akbay et al., 2013). Further studies are required to elucidate how exactly increased telomerase activity mediated by DKC1 overexpression and TP53 inactivation cooperate to promote UCEC formation and progression, or how DKC1 is involved in the UCEC pathogenesis.

The mechanism(s) underlying DKC1 dysregulation in UCEC is incompletely understood, but our findings provide the following clues: (1) Copy number alterations occur frequently in the *DKC1* loci, and copy gains are correlated with their upregulation. However, in tumors carrying DKC1 deletion, DKC1 expression is higher compared with that in tumors with two copies. These results suggest a complicated mechanism for DKC1 regulation. As the *DKC1* gene is localized on X chromosome, one of its alleles is inactivated *via* X-chromosome inactivation (XCI) under physiological conditions. XCI escape aberrantly occurs in oncogenesis (Wang et al., 2020), and thus, the defective XCI is likely attributable to DKC1 dysregulation in UCECs. It is also worth pointing out that DKC1 is expressed from both alleles in female embryo cells (Lansdorp, 2022), and conceivably, such scenario may occur due to de-differentiation of UCEC cells. (2) Sex hormones estrogen and progesterone are known to regulate telomerase activity by inducing and inhibiting TERT transcription, respectively (Wang et al., 2000; Boggess et al., 2006). Our analyses further reveal that MPA significantly inhibited DKC1 expression in UCEC-derived cells, while Estradiol induced strong DKC1 upregulation. These results are conceivable because estrogen and progesterone play a critical role in UCEC pathogenesis (Rodriguez et al., 2019). It is currently unclear whether other factors contribute to DKC1 dysregulation. Nevertheless, DKC1 upregulation promotes telomerase activation in UCEC tumors, as observed in the present study.

Intriguingly, a recent study showed that cancer development mediated by telomere shortening due to mutations in DKC1 or other telomerase-related genes resulted from compromised immune surveillance but not genomic instability (Schratz et al., 2023). Therefore, we analyzed the UCEC immune landscape and its association with DKC1 dysregulation using ESTIMATE, TIDE, TIP and TCIA analyses. The TIDE analysis showed a T Cell exclusion phenotype in DKC1-high tumors, and such immune-cold environment, which was further supported by the TIP evaluation: the diminished trafficking of immune cells into tumor tissues and reduced immune cell infiltration in those tumors. The findings indicate that high DKC1 expression contributes to immune cold tumor environment. On the other hand, DNA repair and mismatch repair (MMR) pathways were highly enriched in DKC1-high tumors, while MMR-proficient UCEC tumors are characterized by immune-cold environments (Ramchander et al., 2019). Consistent with all the above results, DKC1-high tumors exhibited significantly lower sensitivity to immune checkpoint inhibitor (ICI)-based immunotherapy, as assessed using the TCIA tool.

As the H/ACA sno/scaRNP catalytical component, DKC1 pseudouridylates many different RNA molecules (rRNAs, snRNAs, ncRNAs and mRNAs) to regulate ribosome biogenesis, cellular RNA splicing and translation, thereby actively participating in physiological and pathological processes (Angrisani et al., 2014). Indeed, we found the enriched spliceosome in DKC1-high UCEC tumors. However, little has been known about its telomerase-independent effects in oncogenesis. Yoon et al. previously reported that DKC1 inactivation led to impaired translation from internal ribosome entry sites of specific cellular mRNAs, for instance, XIAP and BCL2 mRNAs whose products protected cells from apoptosis (Yoon et al., 2006). It was also shown that DKC1 inhibition resulted in proliferation arrest of neuroblastoma cells via TP53-dependent and independent pathways (O'Brien et al., 2016). Mechanistically, DKC1 depletion caused destabilization of H/ACA snoRNAs and consequent disruption of ribosome biogenesis, eventually inducing a ribosomal stress response (O'Brien et al., 2016). In DKC1-depleted cells, proliferation arrest could not be rescued by TERC, implying that the observed effect was not dependent on telomerase regulation mediated by DKC1. Recent studies unravel that DKC1 promotes proliferation, survival, invasion or metastasis of colon and liver cancer cells by increasing HIF-1α expression and antioxidative effect, respectively (Liu et al., 2012; Ko et al., 2018; Hou et al., 2020). Taken together, DKC1 is actively involved in oncogenesis in telomerase-dependent and independent manners.

Reliable prognostication is important to predict patients at risk for recurrent or metastatic UCEC for personalized therapy and disease surveillance (Abdulfatah et al., 2019; Vizza et al., 2021; Crosbie et al., 2022). In recent years, great efforts have been made to identify new prognostic molecules and to develop molecular classification systems, which have significantly improved the accuracy of UCEC risk stratification (Abdulfatah et al., 2019; Crosbie et al., 2022). As demonstrated in the present study, DKC1 is an independent prognostic factor in UCEC, and contributes to immune-cold microenvironment, which provides a potential biomarker for outcome prediction and precision immunotherapy. Interestingly, the *L1 cell adhesion molecule* (*L1CAM*) gene is very close to the *DKC1* allele on Xq28, and its prognostic role in UCEC has been well characterized (Corrado et al., 2018; Vizza et al., 2020; Giannini et al., 2024). L1CAM drives UCEC aggressiveness by promoting tumor invasion, drug-resistance and metastasis, and therefore in both high- and low-risk diseases, high L1CAM expression is associated with recurrence and/or local and distant metastasis, and poor outcomes (Giannini et al., 2024). Therefore, it may be worth investigating whether DKC1 and L1CAM expression is co-regulated and whether they coordinate to drive UCEC aggressiveness.

## 5 Conclusion

DKC1 dysregulation is widespread in UCEC tumors and may contribute to UCEC pathogenesis through telomerase-dependent and independent pathways. We show that DKC1-high UCEC tumors exhibited an aggressive phenotype featuring higher proliferation, stemness and EMT. This group of tumors are molecularly characterized by higher frequencies of CNAs, HRD, MSI and cancer-driver alterations. T Cell exclusion is the featured microenvironments of DKC1-high UCEC tumors, indicating a poor response to ICI therapy. DKC1-mediated tumor intrinsic and extrinsic mechanisms drive poor patient outcomes. These results are of importance both biologically and clinically and implicated in precision UCEC interventions.

## Data Availability

The original contributions presented in the study are included in the article/Supplementary Material, further inquiries can be directed to the corresponding author.
