# Supplementary material for: Comprehensive analyses of telomerase component DKC1 and its association with clinical, molecular and immune landscapes in uterine corpus endometrial carcinoma
Source: Front Cell Dev Biol. 2025 May 19;13:1592135. doi: 10.3389/fcell.2025.1592135 (PMC12127311; doi:10.3389/fcell.2025.1592135)
Supplement: Supplementary file 1 [file DataSheet1.pdf]

Supplementary Material

Table S1. Clinical characteristics and association with DKC1 expression in 30 UCEC patients.

Variable	Informative number	DKC1 IHC score (mean ± sd)	*P* value
**Age (year)**			0.886
<60	14	2.750 ± 0.3798	
≥60	16	2.719 ± 0.4070	
**Tissue Type**			**0.0007**
Tumor	30	2.733 ± 0.3880	
Normal tissue	12	2.083 ± 0.5967	
**Histology**			0.414
Endometrioid	28	2.714 ± 0.3951	
Serous and mixed	2	3.000 ± 0	
**Stage**			0.081
I	20	2.625 ± 0.4253	
II	5	3.000 ± 0	
III	5	2.900 ± 0.2236	
**FIGO**			**0.014**
1	11	2.500 ± 0.3873	
2	15	2.833 ± 0.3619	
3	4	3.000 ± 0	

Table S2. Clinic-pathological characteristics and association with patients' status in 349 UCEC (GSE2109).

Variable		Informative number	Recurred or progressed		*P* value
			Yes (N) (%)	No (N) (%)	
Age (year)					0.68
	< 60	126	24 (19.0)	102 (81.0)	
	≥60	223	48 (21.5)	175 (78.5)	
Histology					0.024
	Endometrioid	289	53 (18.3)	236 (81.7)	
	Serous and mixed	60	19 (31.7)	41 (68.3)	
Stage					<0.001
	I+II	259	33 (12.7)	226 (87.3)	
	III+IV	90	39 (43.3)	51 (56.7)	

Table S3. Multivariate Analysis of Variance (MANOVA) analysis of DKC1, stage, grade for molecular features and genomic alterations.

Variable	DKC1 (*P* value)	Stage (*P* value)	Grade (*P* value)	DKC1* Stage (*P* value)	DKC1* Grade (*P* value)	Stage*Grade (*P* value)	DKC1* Stage* Grade (*P* value)
Ki67 expression	0.802	0.601	0.378	0.503	0.461	0.811	0.537
Cell cycle score	0.001	<0.001	0.003	0.003	0.057	0.003	0.045
Stemness score	0.834	0.186	0.435	0.479	0.753	0.642	0.528
EMT score	0.268	0.891	0.097	0.586	0.220	0.091	0.731
Aneuploidy score	0.257	0.962	0.922	0.615	0.619	0.176	0.871
Tumor MTDNA copy number	0.041	0.067	0.288	0.056	0.260	0.208	0.227
HRD score	0.061	0.348	0.355	0.099	0.447	0.033	0.593

A

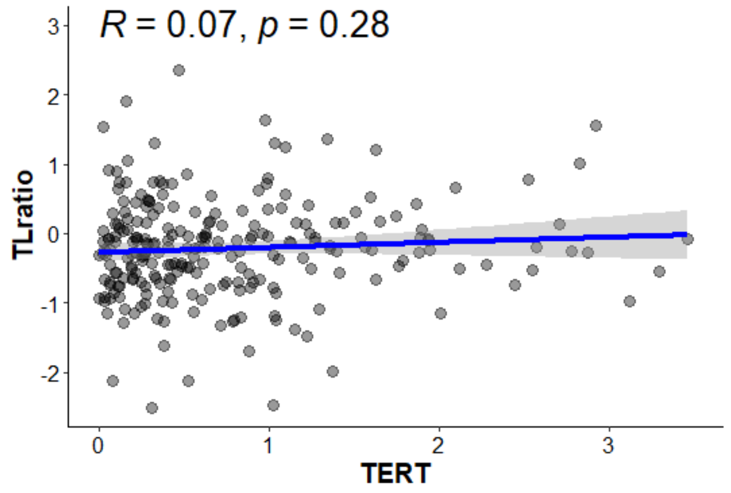

B

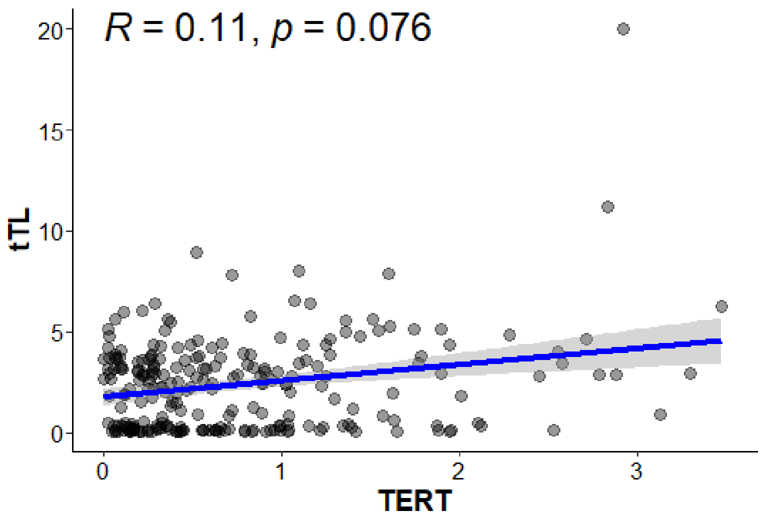

Figure S1. No correlation between TERT expression and the ratios of telomere length of UCEC tumors and corresponding patient blood cells. The TCGA cohort of UCEC was analyzed. (A) TERT mRNA expression is not correlated with telomere length ratio between tumor and blood cell telomere length (TLratio). (B) No correlation between TERT mRNA expression and UCEC tumor telomere length (tTL). Telomere length of UCEC tumors and corresponding patient blood cells were obtained from reference Barthel FP, et al.
